# Effects of Vitamin D_3_, Calcipotriol and FTY720 on the Expression of Surface Molecules and Cytolytic Activities of Human Natural Killer Cells and Dendritic Cells

**DOI:** 10.3390/toxins5111932

**Published:** 2013-10-28

**Authors:** Zaidoon Al-Jaderi, Azzam A. Maghazachi

**Affiliations:** Department of Physiology, Institute of Basic Medical Sciences, Faculty of Medicine, University of Oslo, POB 1103, Oslo N-0317, Norway; E-Mail: zaidoon.al-jaderi@medisin.uio.no

**Keywords:** natural killer cells, dendritic cells, vitamin D_3_, calcipotriol, FTY720, cytotoxicity, receptors, NK17/NK1 cells

## Abstract

We describe here the effects of three drugs that are either approved or have the potential for treating multiple sclerosis (MS) patients through the *in vitro* activities of human natural killer (NK) cells and dendritic cells (DCs). Our results indicate that 1,25(OH)_2_D_3_, the biologically active metabolite vitamin D_3_, calcipotriol and FTY720 augment IL-2-activated NK cell lysis of K562 and RAJI tumor cell lines as well as immature (i) and mature (m) DCs, with variable efficacies. These results are corroborated with the ability of the drugs to up-regulate the expression of NK cytotoxicity receptors NKp30 and NKp44, as well as NKG2D on the surfaces of NK cells. Also, they down-regulate the expression of the killer inhibitory receptor CD158. The three drugs down-regulate the expression of CCR6 on the surface of iDCs, whereas vitamin D_3_ and calcipotriol tend to up-regulate the expression of CCR7 on mDCs, suggesting that they may influence the migration of DCs into the lymph nodes. Finally, vitamin D_3_, calcipotriol and FTY720 enhance NK17/NK1 cell lysis of K562 cells, suggesting that a possible mechanism of action for these drugs is via activating these newly described cells. In conclusion, our results show novel mechanisms of action for vitamin D_3_, calcipotriol and FTY720 on cells of the innate immune system.

## 1. Introduction

Natural killer (NK) cells perform several important functions among them is the regulation of the adaptive immune response by secreting cytokines and chemokines, shaping the innate immune system by interacting with dendritic cells, defending against viral infection, and lysing and destroying tumor cells [1]. In the blood circulation, human NK cells can be classified into two major subsets: those that express CD56 but low CD16 (known as CD56^bright^CD16^low^), and those that express CD16 but low CD56 (known as CD56^dim^CD16^high^) [2].

NK cell development and function are associated with various human diseases [3]. One of these diseases is multiple sclerosis (MS), whereby NK cells play important roles [4,5]. The functional activities of these cells are variable in individuals and are generally lower in MS patients than in healthy individuals [6]. There is a correlation between high mean NK cell activity and total MS lesion load as determined by MRI [7]. As the reduced numbers of NK cells were thought to be mediated by migration into tissues, including the CNS, this could indicate a pathological role of NK cells [8]. In the experimental autoimmune encephalomyelitis (EAE) model, depletion of NK cells resulted in a severe relapsing EAE, and more pronounced CNS pathology [9]. This suggests protective effects for NK cells, especially since the depletion was associated with increased CD4^+^ T cell activity and hence may be associated with direct killing of these cells [10]. Conflicting with these results, it was observed that depletion of NK cells in myelin oligodendrocyte glycoprotein (MOG)-induced EAE actually ameliorated the disease [11]. Additionally, by stimulating NK cells to produce IFN-γ, these cells may also cause inflammation, in part by stimulating a pro-inflammatory Th1 cell response [12]. These findings represent detrimental effects of NK cells eliciting inflammatory lesions and exacerbating the inflammatory response.

One suggested pathway of how NK cells may mediate their effects is by interacting with dendritic cells [4,5]. NK cells have the ability to lyse cells of lymphoid and myeloid lineages, and they kill autologous immature (i) but not mature (m) DCs. The interaction among these cell types takes place at inflammatory sites such as MS lesions. The drug glatiramer acetate (GA), which is approved for treating MS patients, enhanced the cytolysis activity human NK cells against autologous and allogeneic human immature and mature monocyte-derived DCs [13]. Furthermore, administration of GA ameliorated the EAE clinical scores [14], and optimal therapy occurred at time points when NK cells had the highest killing of DCs in EAE mice [14]. Recent observations support these findings in MS patients receiving GA, where the effect of NK cells to lyse DCs in MS patients was monitored for a period of one year. This study reported that dosing MS patients with GA enhanced NK cell lysis of autologous DCs [15]. Collectively, these observations demonstrate the important premise that GA and perhaps other drugs activate NK cells to lyse the professional antigen presenting cells (APCs), consequently impeding antigen presentation and activation of auto-reactive T cells responsible for initiating autoimmune disorders [16].

Vitamin D_3_ deficiency increases the risk of MS, as increased latitude is also correlated with lower blood vitamin D_3_ levels. For instance, ecological studies showed the amount of exposure to sunlight was inversely correlated with the risk of MS, by both regional distribution and as an association with altitude, as well as individual exposure to sunlight [17]. Sunlight is the main source of human vitamin D_3_ through conversion of 7-dehydrocholesterol to previtamin D_3_ in the skin, and through further metabolic steps to active hormone 1,25-dihydroxyvitamin D_3_ [18]. Dietary vitamin D_3_ intake may reduce the risk of MS in spite of latitude-dependent deficiency, for instance in areas where higher amounts of vitamin D_3_-rich fish are consumed [19]. There is also an association with disease in the EAE model, as dosing of vitamin D_3_ prevented the disease [20,21]. Definite effects of supplementing patients with vitamin D_3_ have not yet been shown, but some studies indicate that serum concentrations of vitamin D_3_ may affect disease severity [18]. It was also observed that MS patients receiving vitamin D_3_ have less relapses than control groups [22]. Also, increased serum level of vitamin D_3_ in MS patients resulted in improved T regulatory (Treg) cell activity, corroborated with suppression of auto-reactive T cells and a switch from a Th1 to Th2 phenotype [23]. This was later supported by the same authors who showed an increase in the proportion of IL-10-secreting T cells after supplementing MS patients with vitamin D_3_ [24]. In cuprizone-fed animals, supplementation with this vitamin protected these animals from demyelination associated with reduced microglia activation and macrophage infiltration [25].

On the other hand, the drug FTY720 “fingolimod; 2-amino-2-(2-[4-octylphenyl]ethyl)-1,3-propanediol)” is an immunosuppressive drug derived from myriocin, a fungal metabolite that resembles sphingosine. Its mechanism of action is related to binding four out of five sphingosine-1-phosphate (S1P) receptors, namely S1P_1_, S1P_3_, S1P_4_ and S1P_5_, and particularly S1P_1_ resulting in its internalization with a consequent inhibition of S1P activity [26]. IL-2-activated NK cells express S1P_1,3,4,5_, and S1P induced the *in vitro* chemotaxis of these cells [27]. It was also reported that S1P inhibited NK cell lysis of target cells including tumor cells and DCs [28,29], and that FTY720 reversed this inhibitory activity [29].

In accordance with this rational and due to the roles NK cells or DCs play in MS and other autoimmune diseases, the present study was conducted to examine the effects of drugs such as vitamin D_3_, its analog calcipotriol, and FTY720, which are either already approved or have potential for treating MS patients, on the expression of surface molecules in these cells. In addition, this paper also examines the effects of the drugs on NK cell lysis of tumor cells and dendritic cells.

## 2. Results

### 2.1. Effects of the Drugs on NK Cell Lysis of Target Cells

**Figure 1:**
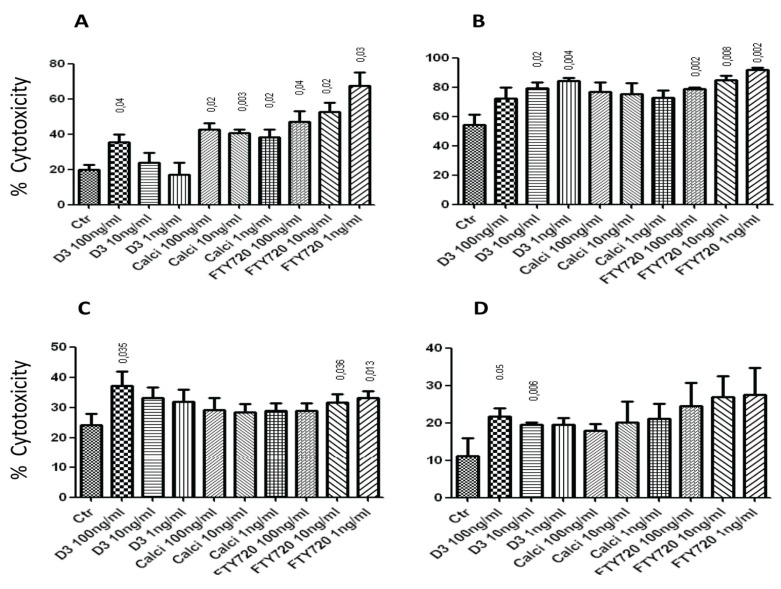
(**A**) Various concentrations of 1,25(OH)_2_D_3_, calcipotriol and FTY720 augment NK cells lysis of K562 target cells. E:T cell ratio shown is 2:1. (**B**) 1,25(OH)_2_D_3_ and FTY720 significantly enhance NK cells killing of RAJI target cells; E:T ratio is 10:1. (**C**) 1,25(OH)_2_D_3_ and FTY720 significantly augment NK cells cytolysis of immature DCs. E:T ratio is 10:1. (**D**) Effects of 1,25(OH)_2_D_3_, calcipotriol and FTY720 on NK cells lysis of mature DCs. E:T ratio is 10:1. In all experiments, NK cells were pre-treated with the drugs for 4 h at 37 °C, washed and then incubated with the target cells. Mean ± SEM of four or five experiments performed. *p* values comparing the effects of the drugs to the control (Ctr; no drug added) are shown on top of the columns.

The first set of experiments attempted to show whether 1,25(OH)_2_D_3_, calcipotriol or FTY720 have any effect on NK cell lysis of tumor cells or dendritic cells (DCs). Results in Figure 1A show that 100 ng/mL of 1,25(OH)_2_D_3_, as well as the 1, 10 and 100 ng/mL concentrations of calcipotriol and FTY720 significantly enhanced NK cell lysis of K562 tumor target cells. In these experiments, several effector:target (E:T) cell ratios were used, but only the 2:1 E:T ratio is shown in the figure. Similarly, 10 and 1 ng/mL of 1,25(OH)_2_D_3_ significantly enhanced NK cell lysis of RAJI tumor cells (Figure 1B). Also, all three concentrations of calcipotriol increased such activity, but this was not statistically significant. However, the 1, 10 or 100 ng/mL of FTY720 significantly augmented NK cell killing of RAJI cells (Figure 1B).

The 100 ng/mL of 1,25(OH)_2_D_3_ augmented NK cell lysis of immature DCs “iDCs” but there was no effect for calcipotriol, whereas FTY720 increased NK cell lysis of these cells (Figure 1C). Similarly, the 100 and 10 ng/mL concentrations of 1,25(OH)_2_D_3_ significantly enhanced NK cell killing of mature DCs “mDCs” (Figure 1D). Although calcipotriol and FTY720 showed increased NK cell killing of mDCs, this did not reach statistical significance (Figure 1D). In summary, it appears that 1,25(OH)_2_D_3_, calcipotriol and FTY720 augment NK cell lysis of tumor target cells, as well as iDCs and mDCs with variable efficacies. The lack of dose response in some of these findings could be due to variations among individual blood samples. It may also be due to seasonal changes as NK cells may respond differently during the summer than during the winter. Another variable could be the time of obtaining the blood samples due to changes in the levels of cortisol and other hormones which affect NK cells, from morning to evening.

### 2.2. 1,25(OH)_2_D_3_, Calcipotriol and FTY720 Up-Regulate the Expression of NK Cytotoxicity Molecules on the Surface of NK Cellstitle

**Figure 2:**
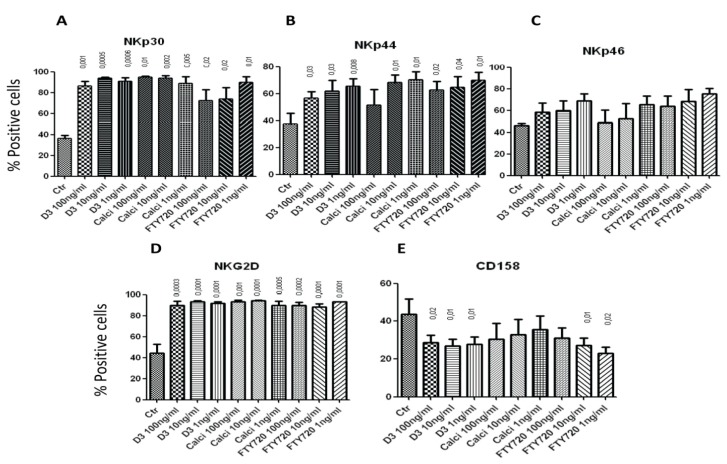
Treatment of NK cells with drugs modulates their expression of NK cytotoxicity receptors or KIR. Human NK cells were pre-treated with 100, 10 or 1 ng/mL of 1,25(OH)_2_D_3_ calcipotriol or FTY720 for 4 h, washed and the expression of cytotoxicity receptors NKp30 (**A**), NKp44 (**B**) or NKp46 (**C**), as well as NKG2D (**D**) was determined by flow cytometric analysis. (**E**) The expression of CD158 was also examined. Mean ± SEM of four or five experiments performed. *p* values comparing the effects of the drugs to the control (Ctr; no drug added) are shown on top of the columns. Shown are percentages of positive cells expressing the particular marker. A similar pattern was observed when mean fluorescence intensity (MFI) was examined (not shown).

To correlate the increase of cytotoxicity with the ability of the drugs to modulate the expression of NK natural cytotoxicity receptors, we examined such expression after incubation with the drugs for 4 h. All three concentrations of 1,25(OH)_2_D_3_, calcipotriol and FTY720 significantly up-regulated the expression of NKp30 on the surface of NK cells after 4 h incubation (Figure 2A). Similarly, 1,25(OH)_2_D_3_, calcipotriol or FTY720 up-regulated the expression of NKp44 on the surface of these cells (Figure 2B). Although there was a trend of increased expression of NKp46 after incubating NK cells for 4 h with the drugs, this increase was not significant (Figure 2C). However, the 1, 10 and 100 ng/mL of 1,25(OH)_2_D_3_, calcipotriol and FTY720 significantly augmented the expression of NKG2D on the surface of NK cells after 4 h incubation (Figure 2D). Interestingly, the three concentrations of 1,25(OH)_2_D_3_ reduced the expression of the killer inhibitory molecule (KIR) CD158 (Figure 2E). FTY720 exerted almost similar effect (Figure 2E).

### 2.3. Effect of the Drugs on the Cytolytic Activity of NK17/NK1 Cells

**Figure 3:**
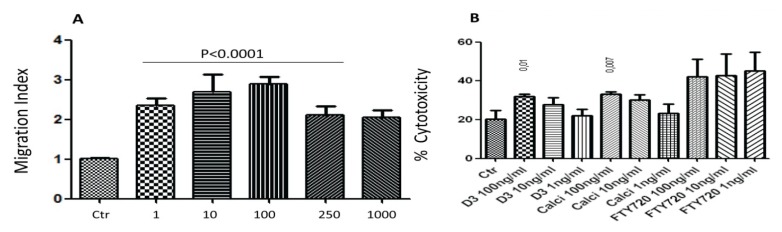
NK17/NK1 cell (CD56^+^CCR4^+^) chemotaxis and cytolytic activity. (**A**) CD56^+^ NK cells were placed in the upper wells of Boyden chambers, whereas 1, 10, 100, 250 and 1000 ng/mL of MDC/CCL22 chemokine were placed in the lower wells. After 2 h, the filters were collected, stained with Giemsa stain and the cells were counted. Migration Index was calculated as the number of cells migrating towards the chemokine divided by the number of cells migrating towards media only (Ctr; control). Mean ± SEM of four experiments performed, in each experiment five or six filters were used and the number of cells was averaged. *p* values comparing the effects of the drugs to the control (Ctr; no drug used) are shown on top of the columns. (**B**) NK17/NK1 cells were pre-treated with 100, 10 or 1 ng/mL of 1,25(OH)_2_D_3_, calcipotriol or FTY720 for 4 h, washed and incubated with K562 cells at 10:1 E:T ratio. Mean ± SEM of three experiments performed. *p* values comparing the effects of the drugs to the control (Ctr; no drug added) are shown on top of the columns.

We recently described the existence of new cells, named NK17/NK1 cells which express CD56 and CCR4 [30]. To support these findings, we examined whether CD56^+^ NK cells migrate towards concentrations gradient of MDC/CCL22, the ligand for CCR4 [31]. In the *in vitro* chemotaxis experiments, CD56^+^ NK cells were placed in the upper compartments of modified Boyden chambers, whereas various concentrations of MDC/CCL22 were placed in the lower compartments. After 2 h, the cells that migrated towards the chemokine were counted. Results in Figure 3A show a typical bell-shape curve of chemotactic response for these cells; all concentrations of MDC/CCL22 examined induced *in vitro* chemotaxis (*p* < 0.0001).

Further, we investigated whether various drugs affect the cytolytic activity of NK17/NK1 cells against K562 cells. These cells were isolated based on the surface expression of CD56 and CCR4. At 10:1 E:T cell ratio, NK17/NK1 cells kill almost at the same rate as the 2:1 E:T cell ratio of heterogeneous CD56^+^ NK cells, suggesting that they are less potent killers than other subsets of NK cells (compare results in Figure 3B with Figure 1A). What is important is the enhancement of NK17/NK1 cell lysis against K562 cells after incubation with 100 ng/mL of 1,25(OH)_2_D_3_ or calcipotriol (Figure 3B). The three concentrations, *i.e*., 1, 10 or 100 ng/mL of FTY720 also increased their killing potential, though this was not statistically significant (Figure 3B).

### 2.4. Effects of Incubating iDCs with the Drugs on the Expression of Surface Molecules

**Figure 4:**
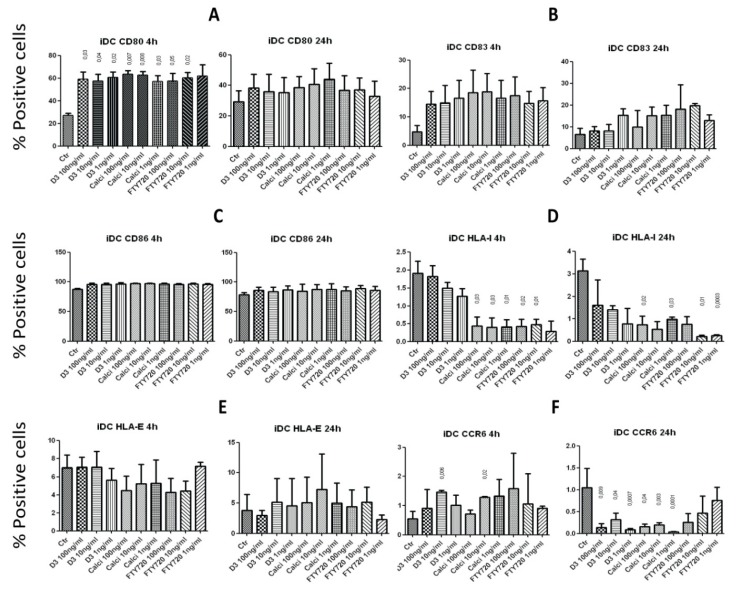
Treatment of immature DCs with the drugs modulates their surface expression. Immature DCs were incubated for 4 h or 24 h with 100, 10 or 1 ng/mL of 1,25(OH)_2_D_3_, calcipotriol or FTY720, washed and then examined. The expression of CD80 (**A**), CD83 (**B**), CD86 (**C**), HLA-I (**D**), HLA-E (**E**) and the chemokine receptor CCR6 (**F**) was investigated. *p* values comparing the effects of the drugs to the control (Ctr; no drug added) are shown on top of the columns. Shown are percentages of positive cells expressing the particular marker. A similar pattern was observed when mean fluorescence intensity (MFI) was examined (not shown).

To investigate whether the drugs affect iDCs, we sought to determine the expression of CD80, CD83 and CD86, as well as MHC molecules HLA-I and HLA-E and the chemokine receptor CCR6, after incubating iDCs with various concentrations of the drugs for 4 and 24 h. After 4 h incubation, the 1, 10 and 100 ng/mL concentrations of 1,25(OH)_2_D_3_, calcipotriol and FTY720 significantly up-regulated the expression of the co-stimulatory molecule CD80 on iDCs, but this effect disappeared after 24 h incubation (Figure 4A). In contrast, none of the drugs significantly affected the expression of CD83 or CD86 after 4 or 24 h incubation with iDCs (Figure 4B,C). Calcipotriol and FTY720 down-regulated the expression of HLA-I on the surface of iDCs after 4 h incubation, and this activity persisted after 24 h incubation (Figure 4D). However, none of the drugs examined affected the expression of HLA-E on the surface of iDCs after 4 or 24 h incubation (Figure 4E). Surprising results were observed when the expression of the chemokine receptor CCR6 was investigated after incubation with the drugs; whereas the 10 ng/mL concentration of 1,25(OH)_2_D_3_ and calcipotriol increased the percentages of CCR6 expressing cells after 4 h incubation with iDCs, all three concentrations of these two drugs down-regulated such activity after 24 h incubation (Figure 4F).

### 2.5. Effects on the Expression of Surface Molecules on mDCs

**Figure 5:**
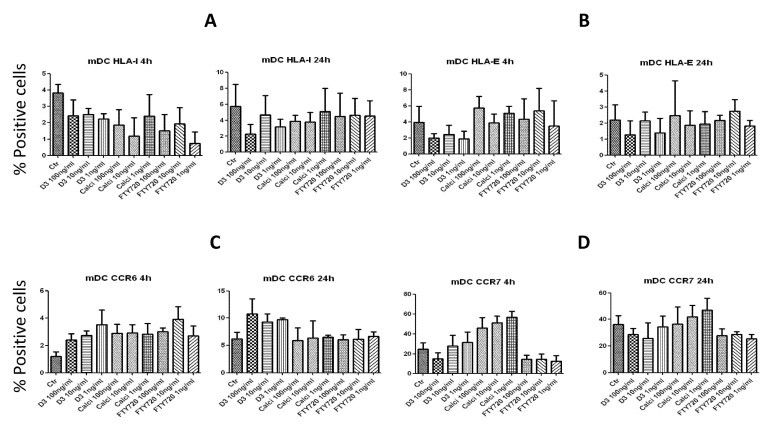
Treatment of mature DCs with the drugs modulates their surface expression. Mature DCs were incubated for either 4 h or 24 h with 100, 10 or 1 ng/mL of 1,25(OH)_2_D_3_, calcipotriol or FTY720, washed and then examined. The expression of HLA-I (**A**), HLA-E (**B**), or the chemokine receptors CCR6 (**C**) and CCR7 (**D**) was investigated. Shown are percentages of positive cells expressing the particular marker. A similar pattern was observed when mean fluorescence intensity (MFI) was examined (not shown).

We next investigated whether the drugs may modulate the expression of mDCs surface molecules. To perform these experiments, mDCs were incubated with three different concentrations of 1,25(OH)_2_D_3_, calcipotriol or FTY720 for 4 h or 24 h, and the expression of various molecules was then examined. Because there was absolutely no effects of any of the drugs on the expression of CD80, CD83 or CD86 after incubation with mDCs for 4 or 24 h, these results were not shown. Also, there was no significant effects on the expression of HLA-I molecule (Figure 5A), HLA-E molecules (Figure 5B), or CCR6 (Figure 5C). However, there was a tendency of up-regulating the chemokine receptor CCR7 after incubation with 1,25(OH)_2_D_3_ or calcipotriol for 4 h, but this increase did not reach significance (Figure 5D).

## 3. Discussion

Vitamin D_3_ affects various disorders, including asthma and chronic obstructive pulmonary disease [32]. In addition to multiple sclerosis [22,23,24], Vitamin D_3_ is involved in ameliorating other autoimmune diseases [33]. Further, vitamin D_3_ is critical for protecting against experimental autoimmune prostatitis [34]. Although several mechanisms of action were described for vitamin D_3_ such as a Th1/Th2 switch, or the activation of T-regulatory cells [23,24], the effects of vitamin D_3_ on NK cells or DCs have not been studied in details. NK cells play important roles in MS, and whether they ameliorate or exacerbate the disease is still a matter of discussion [4,5]. It was previously reported that vitamin D_3_ prevents the generation of IFN-DCs when added to freshly isolated monocytes, and is capable of redirecting already differentiated IFN-DCs towards a more immature stage. In the same study, it was observed that the chemotactic response of IFN-DCs to CCL4 and CCL19 was markedly reduced or completely abrogated by vitamin D_3_, revealing a novel mechanism involved in vitamin D_3_-mediated immunomodulation [35]. Lee *et al*. showed that vitamin D_3_ activates NK cells to kill melanoma cells, an activity related to FAS up-regulation and induction of apoptosis [36]. In another study it was shown that NK cells by releasing IL-8 recruit eosinophils in allergic rhinitis, an effect that is inhibited by vitamin D_3_, indicating that this vitamin may play an important role as an anti-inflammatory molecule [37].

In the present study, we observed that the biologically active metabolite of vitamin D_3_ (1,25(OH)_2_D_3_), calcipotriol and FTY720 augment IL-2-activated NK cell lysis of tumor target cells K562 and RAJI with variable efficacies. In addition, vitamin D_3_ activates these cells to lyse both iDCs and mDCs. We correlated this cytolytic activity with the ability of these drugs to up-regulate the expression of NK cytotoxicity receptors. Vitamin D_3_, calcipotriol and FTY720 enhance the expression of NKp30, NKp44 and NKG2D on the surface of these cells, whereas NKp46 expression is not significantly up-regulated. NKp30 plays important roles in NK cell killing of tumor cells through its binding to B7-H6 ligand expressed by tumor cells [38]. It is also involved in NK cell lysis of iDCs by binding to BAG6 molecule expressed on iDCs [39]. NKp44, on the other hand, is up-regulated on NK cells upon activation [40], and has a distinct similarity to NKp30 since they both are encoded by the same locus [41]. Although the tumor target molecule that binds NKp46 has not yet been described, it was suggested that glycosylation of tumor cells may lead to recognition by NKp46 molecule [42]. The other molecule that is up-regulated by the drugs is type II transmembrane protein NKG2D, which recognizes MHC class I chain-related molecules (MICA and MICB), as well as UL16-binding proteins (ULPBs) that are expressed on stressed cells, including tumor cells [43]. Collectively, it can be suggested that one mechanism of action for vitamin D_3_, calcipotriol and FTY720 in ameliorating diseases is perhaps through up-regulating the expression of NK cell cytotoxicity molecules such as NKp30 and NKp44, as well as NKG2D on the surface of NK cells. In addition, vitamin D_3_ and FTY720 down-regulate the killer inhibitory receptor CD158, which may add to NK cell cytolytic activity.

We also observed that the three drugs described here down-regulate the expression of HLA-I after both 4 and 24 h of incubation with iDCs. These results suggest that vitamin D_3_, calcipotriol and FTY720 may impede presentation of antigens to T cells. In contrast, no effect of any of the drugs was observed on mDCs. Raich-Regue *et al*. reported the generation of tolerogenic DCs from MS patients after incubation with vitamin D_3_ [44]. These cells down-regulate the expression of CD83, CD86 and HLA-DR among other molecules after incubation with vitamin D_3_. As indicated, the only molecule that is down-regulated on iDCs is HLA-I. In addition, we observed reduced expression of CCR6 in iDCs after 24 h incubation with vitamin D_3_ or calcipotriol, and a tendency of increased expression of the chemokine receptor CCR7 on the surface of mDCs by the same drugs, thereby suggesting that vitamin D_3_ or calcipotriol may encourage mDCs to migrate into secondary lymphoid tissues and, in particular, the peripheral lymph nodes.

A Phase II clinical trial using FTY720 involving 255 MS patients with relapsing-remitting MS (RRMS) patients showed a reduction in the median total number MS lesions [45]. MS patients who participated in FTY720 therapy protocol showed clear reductions in annualized relapse rates and lesion counts compared with the placebo patients during a two-year period [46]. Consequently, this drug has been recently approved for treatment of MS patients under the name Gilenya (Novartis Pharma AG, Basel, Switzerland). We previously reported that FTY720 reversed the inhibitory effect of S1P on NK cell lysis of tumor target cells or DCs [29]. This effect of FTY720 was attributed to binding SIP_1_ on the surface of NK cells. However, the present report describes a direct effect of FTY720 on NK cell lysis of K562, RAJI, iDCs and mDCs. This activity could be due to binding SIP_1_, but in this case FTY720 acts as an agonist. Alternatively, FTY720 may exert its effect by inhibiting the action of sphingosine kinase 1 (SK1) which generates the inhibitory molecule S1P, or could be due to other activities described for FTY720 [47].

Finally, we examined the effects of the three drugs on the cytolytic activity of NK17/NK1 cells. These are newly described cells that secrete IL-17 and IFN-γ, express the CD56 and CCR4 surface molecules, as well as the transcription factors RORγ and T-bet [30]. They are abundant in the cerebrospinal fluid (CSF) of MS patients but their functions are not yet understood. Here we report that these cells lyse tumor target cells, albeit with less efficacy than heterogeneous T cells. Furthermore, we observed that vitamin D_3_, calcipotriol and FTY720 potentiate their lysis of target cells. These results suggest that NK17/NK1 cells may have important activity in the CSF of MS patients, although this was not examined in this report. Of note, these cells are also generated from the blood of normal or MS patients after activation with IL-2 [30]. Hence, they may participate in lysing target cells under pathological conditions and during inflammation or at tumor growth sites. These presumed activities of NK17/NK1 cells are currently under investigation.

## 4. Experimental Section

### 4.1. Cell Preparations

Buffy coats of healthy volunteers were obtained from the blood bank (Ullevål Hospital, Oslo, Norway). NK cells were purified using RosetteSep NK Cell enrichment cocktail (Stem Cell technologies SARL, Grenoble, France), which depletes CD3, CD4, CD19, CD36, CD66b, CD123 and glycophorin A on RBCs leaving pure NK cells intact. NK cells were collected, washed and cultured at 1 × 10^6^/mL with 200 U/mL IL-2 (Peprotech, Rocky Hill, NJ, USA) at 37 °C in a 5% CO_2_ incubator. IL-2 (200 U/mL) was added to the culture after 2, 4 and 6 days. NK cells were collected after 7 days, washed and then separated into CD56^+^ and CD56^−^. Viability was more than 90% after the incubation period as determined by trypan blue exclusion test.

Human monocytes were isolated using RosetteSep Human monocyte enrichment cocktail (Stem Cell technologies Europe, Grenoble, France), which depletes CD2, CD3, CD8, CD19, CD56, CD66b and glycophorin A on RBCs leaving CD14^+^ monocyte cells intact. CD14^+^ cells were cultured in sterile Petri dishes at 1.5 × 10^6^/mL with 6 ng/mL IL-4 and 25 ng/mL rhGM-CSF (PeproTech, London, UK) for 5 days at 37 °C in a 5% CO_2_ to generate immature dendritic cells (iDCs). Mature dendritic cells (mDCs) were generated by adding 1 µg/mL LPS (Sigma-Aldrich, Oslo, Norway) to the cultures of iDCs for 2 days, as previously described [48].

### 4.2. Isolation of NK17/NK1 Cells

IL-2-activated NK cells were sorted into CD56^+^ and CD56^−^ by magnetic separation using EasySep Human CD56 Positive selection kit (Stem Cell technologies). To isolate CD56^+^ cells, NK cells were resuspended in RoboSep-buffer (PBS without Ca and Mg, 2% BSA and 1 mM EDTA) (Sigma-Aldrich, Oslo, Norway), at 1 × 10^8^ cells/mL and incubated with EasySep Human CD56 Positive selection kit (Stem Cell technologies Europe, Grenoble, France), for 15 min at room temperature. A 50 µL/mL of magnetic nanoparticle was then added and incubated for 10 min at room temperature. Afterwards, the mixture was resuspended into 2.5 mL by adding RoboSep-buffer and placed into the magnet for 5 min. CD56^+^ cells were collected, counted, and checked for viability. To isolate CCR4^+^ cells, CD56^+^ cells (1 × 10^7^ cells/mL) were incubated with pre-coated Dynabeads with mouse anti-human CCR4 (R&D Systems Europe, Ltd., Abingdon, UK). Recovered CCR4^+^ CD56^+^ cells were collected and examined as described [30]. These cells were examined by flow cytometry for the production of IL-17 and IFN-γ before being used in the chemotaxis or the cytotoxicity assays.

### 4.3. Cytotoxicity Assay

NK cells were incubated at 1 × 10^6^ cells/mL with or without 100, 10 or 1 ng/mL of vitamin D_3_ (1α, 25-Dihydroxyvitamin D_3_; Sigma-Aldrich), calcipotriol (Calcipotriol hydrate; Sigma-Aldrich) or FTY720 (a generous gift from Dr. Volker Brinkmann, Novartis Pharma AG, Basel, Switzerland) for 4 h and then used as effectors cells against the human myeloid leukemia cell line K562 cells (CCL-243 obtained from American Type Culture Collection “ATCC”, Manassas, VA, USA) or RAJI human lymphoma cells (CCL-86, ATCC). Autologous iDCs or mDCs were also used as target cells. These target cells were incubated at 1 × 10^6^ cells/mL with 5 µg/mL calcein-AM (Sigma-Aldrich) for 1 h at 37 °C, washed and plated onto 96-well flat bottom plates, with effectors cells at various E:T ratios in triplicates. To obtain total killing, target cells were incubated with 0.5% Triton-X (Sigma-Aldrich) for the last 30 min of incubation, whereas total viability was obtained by incubating the cells with media only. The fluorescence intensity of the calcein AM-loaded target cells was measured with BioTek FLX 800 plate reader (Bio-Tek Instruments Inc., Winooski, VT, USA), using 485/528 nm fluorescence filters. The percentage of cytotoxicity was calculated according to the following formula: % Viability = fluorescence units (FU) of targets incubated with NK cells (experimental), minus FU of targets incubated with Triton-X (total lysis), divided by FU of targets incubated with media only (total viability), minus FU of targets incubated with Triton-X (total lysis). Percent cytotoxicity was then calculated as 100% minus % viability.

### 4.4. Flow Cytometric Analysis

IL-2-activated NK cells were incubated with various drugs for 4 h at 37 °C. After this period, cells were washed and resuspended at 0.3 × 10^6^ in FACS-buffer (PBS without Ca and Mg, 2% FBS and 10 mM NaN_3_) (Sigma-Aldrich), and labeled in the dark for 45 min at 4 °C with 2 µg/mL FITC-conjugated mouse anti-human CCR4, FITC-conjugated mouse IgG2B isotype control, PE-conjugated mouse anti-human NKG2D (CD314), PE-conjugated mouse anti-human NKp30 (CD337), PE-conjugated mouse anti-human NKp44 (CD336), PE-conjugated mouse anti-human NKp46 (CD335), PE-conjugated mouse anti-human CD56, PE-conjugated mouse IgG1 isotype control, PE-conjugated mouse anti-human KIR/CD158, or PE-conjugated mouse IgG2B isotype control (these antibodies were purchased from BD pharmingen, San Diego, CA, USA). Immature DCs and mDCs were incubated for 4 or 24 h with various drugs and then labeled with FITC-conjugated mouse anti-human CCR6, FITC-conjugated mouse IgG2B isotype control, FITC-conjugated mouse anti-human CCR7, FITC-conjugated mouse IgG2A isotype control, FITC-conjugated mouse anti-human-B7-1/CD80, FITC-conjugated mouse anti-human-B7-2/CD86, FITC-conjugated mouse anti-human CD83, or FITC-conjugated Mouse IgG1 isotype control (these antibodies were obtained from R&D systems, Abingdon, UK). The cells were washed twice, resuspended with FACS buffer and analyzed in flow cytometry (FACS Calibur or FACS canto II, Beckton Dickinson Biosciences, San Jose, CA, USA).

For three-color analysis, cells were first labeled with surface markers, fixed with 4% paraformaldehyde for 20 min, and then washed twice with SAP buffer (PBS without Ca and Mg, 0.1% Saponin and 0.05% NaN_3_) (Sigma-Aldrich, Oslo, Norway). The cells were stained intracellularly with PE-conjugated mouse anti-human IFN-γ, PE-conjugated mouse IgG2B isotype control, APC-conjugated mouse anti-human IL-17, or APC-conjugated mouse IgG1 isotype control (all antibodies were from R&D systems) for 30 min in the dark at 4 °C. Stained cells were washed twice and medium was replaced with FACS buffer and analyzed in the flow cytometry. Only those CD56^+^CCR4^+^ that secrete IL-17 and IFN-γ (NK17/NK1 cells) were collected and analyzed further.

For labeling with other antibodies, cells were stained with purified mouse anti-human HLA-class I, purified mouse anti-human HLA-E, purified mouse IgG1κ isotype control (eBioscience, Inc., San Diego, CA, USA) in the dark for 45 min at 4 °C, washed twice and incubated in the dark for another 45 min at 4 °C with FITC-conjugated goat anti-mouse IgG (BD pharmingen, San Diego, CA, USA). Stained cells were washed twice, and medium was replaced with FACS buffer and analyzed in the flow cytometry. Compensation was done according to the isotype control. Analysis was done by FlowJo (Flo cytometry analysis software, Ashland, OR, USA).

### 4.5. Chemotaxis Assay

CD56^+^ NK cells (5 × 10^5^) were placed in the upper wells of modified Boyden chambers, whereas 1,000, 250, 100, 50 and 10 ng/mL MDC/CCL22 were placed in the lower chambers. The cells were separated from the chemokine by 8 µm Nuclepore membranes (Whatman International Ltd., Kent, UK). After 2 h of incubation at 37 °C, the filters were removed, dehydrated, stained with 15% modified Giemsa stain for 7 min, and then mounted on glass slides. Cells in high-power fields were counted and averaged for each sample. Migration index (MI) was calculated as the number of cells migrating towards the concentration gradients of MDC/CCL22 divided by the number of cells migrating towards medium only.

### 4.6. Statistical Analysis

Significant values were generated using Student’s *t*-test calculated by Graphpad Prism program (San Diego, CA, USA)

## 5. Conclusions

We observed novel mechanisms of action for the biologically active metabolite of vitamin D_3_, its analog calcipotriol and FTY720, drugs either approved or with the potential for treating autoimmune diseases. All three drugs examined augment *in vitro* IL-2-activated NK cell lysis of K562 and RAJI tumor cell lines, as well as immature and mature DCs, with variable efficacies. The observations described here may be potentially used for treating patients with MS or other autoimmune diseases.

## References

[1] Fauriat C., Long E.O., Ljunggren H.G., Bryceson Y.T. (2010). Regulation of human NK-cell cytokine and chemokie production by target cell recognition. Blood.

[2] Maghazachi A.A. (2005). Compartmentalization of human natural killer cells. Mol. Immunol..

[3] Wood S.M., Ljunggren H.G., Bryceson Y.T. (2011). Insights into NK cell biology from human genetics and disease associations. Cell. Mol. Life Sci..

[4] Maghazchi A.A. (2013). On the role of natural killer cells in neurodegenerative diseases. Toxins.

[5] Maghazachi A.A. (2012). Role of natural killer cells in multiple sclerosis. ISRN Immunol..

[6] Benczur M., Petranyl G.G., Palffy G., Varga M., Talas M., Kotsy B., Földes I., Hollán S.R. (1980). Dysfunction of natural killer cells in multiple sclerosis: A possible pathogenetic factor. Clin. Exp. Immunol..

[7] Kastrukoff L.F., Morgan N.G., Aziz T.M., Zecchini D., Berkowitz J., Paty D.W. (1998). Natural killer (NK) cells in chronic progressive multiple sclerosis patients treated with lymphoblastoid interferon. J. Neuroimmunol..

[8] Grunebaum E., Malatzky-Goshen E., Shoenfeld Y. (1989). Natural killer cells and autoimmunity. Immunol. Res..

[9] Zhang B., Yamamura T., Kondo T., Fujiwara M., Tabira T. (1997). Regulation of experimental autoimmune encephalomyelitis by natural killer (NK) cells. J. Exp. Med..

[10] Kaur G., Trowsdale J., Fugger L. (2013). Natural killer cells and their receptors in multiple sclerosis. Brain.

[11] Winkler-Pickett R., Young H.A., Cherry J.M., Diehl J., Wine J., Back T., Bere W.E., Mason A.T., Ortaldo J.R. (2008). *In vivo* regulation of experimental autoimmune encephalomyelitis by NK cells: Alteration of primary adaptive responses. J. Immunol..

[12] Vollmer T.L., Liu R., Price M., Rhodes S., La C.A., Shi F.D. (2005). Differential effects of IL-21 during initiation and progression of autoimmunity against neuroantigen. J. Immunol..

[13] Sand K.L., Rolin J., Knudsen E., Al-Falahi Y., Maghazachi A.A. (2009). Modulation of natural killer cell cytotoxicity and cytokine release by the drug glatiramer acetate. Cell. Mol. Life Sci..

[14] Al-Falahi Y., Sand K.L., Knudsen E., Damaj B.B., Rolin J., Maghazachi A.A. (2009). Splenic natural killer cell activity in two models of experimental neurodegenerative diseases. J. Cell. Mol. Med..

[15] Høglund R., Harbo H.F., Holmøy T., Maghazachi A.A. (2013). A one year follow-up study of natural killer and dendritic cells activities in multiple sclerosis patients receiving glatiramer acetate. PLoS One.

[16] Hestvick A.L.K. (2010). The Double-edged sword of autoimmunity: Lessons from multiple sclerosis. Toxins.

[17] Ascherio A., Munger K.L. (2007). Environmental risk factors for multiple sclerosis. Part II: Noninfectious factors. Ann. Neurol..

[18] Ascherio A., Munger K.L., Simon K.C. (2010). Vitamin D and multiple sclerosis. Lancet Neurol..

[19] Brustad M., Sandanger T., Aksnes L., Lund E. (2004). Vitamin D status in a rural population of northern Norway with high fish liver consumption. Public Health Nutr..

[20] Lemire J.M., Archer D.C. (1991). 1,25-dihydroxyvitamin D_3_ prevents the *in vivo* induction of murine experimental autoimmune encephalomyelitis. J. Clin. Investig..

[21] Cantorna M.T., Hayes C.E., DeLuca H.F. (1996). 1,25-Dihydroxyvitamin D_3_ reversibly blocks the progression of relapsing encephalomyelitis, a model of multiple sclerosis. Proc. Natl. Acad. Sci. USA.

[22] Burton J.M., Kimball S., Vieth R., Bar-Or A., Dosch H.M., Cheung R., Gagne D., D’Souza C., Ursell M., O’Connor P. (2010). A phase I/II dose-escalation trial of vitamin D_3_ and calcium in multiple sclerosis. Neurology.

[23] Smolders J., Thewissen M., Peelen E., Menheere P., Tervaert J.W., Damoiseaux J., Hupperts R. (2009). Vitamin D status is positively correlated with regulatory T cell function in patients with multiple sclerosis. PLoS One.

[24] Smolders J., Peelen E., Thewissen M., Tervaert J.W., Menheere P., Hupperts R., Damoiseaux J. (2010). Safety and T cell modulating effects of high hose vitamin D_3_ supplementation in multiple sclerosis. PLoS One.

[25] Wergeland S., Torkildsen Ø., Myhr K.M., Aksnes L., Mørk S.J., Bø L. (2011). Dietary vitamin D3 supplements reduce demyelination in the cuprizone model. PLoS One.

[26] Brinkmann V. (2007). Sphingosine 1-phosphate receptors in health and disease: Mechanistic insights from gene deletion studies and reverse pharmacology. Pharmacol. Ther..

[27] Kveberg L., Bryceson Y., Inngjerdingen M., Rolstad B., Maghazachi A.A. (2002). Sphingosine 1 phosphate induces the chemotaxis of human natural killer cells. Role for heterotrimeric G proteins and phosphoinositide 3 kinases. Eur. J. Immunol..

[28] Lagadari M., Lehmann K., Ziemer M., Truta-Feles K., Berod L., Idzko M., Barz D., Kamradt T., Maghazachi A.A., Norgauer J. (2009). Sphingosine-1-phosphate inhibits the cytotoxic activity of NK cells via Gs protein-mediated signaling. Int. J. Oncol..

[29] Rolin J., Sand K.L., Knudsen E., Maghazachi A.A. (2010). FTY720 and SEW2871 reverse the inhibitory effect of S1P on natural killer cell mediated lysis of K562 tumor cells and dendritic cells but not on cytokine release. Cancer Immunol. Immnuother..

[30] Pandya A.D., Al-Jaderi Z., Høglund R.A., Holmøy T., Harbo H.F., Norgauer J., Maghazachi A.A. (2011). Identification of human NK17/NK1 cells. PLoS One.

[31] Godiska R., Chantry D., Raport C.J., Sozzani S., Allavena P., Leviten D., Mantovani A., Gray P.W. (1997). Human macrophage-derived chemokine (MDC), a novel chemoattractant for monocytes, monocyte-derived dendritic cells, and natural killer cells. J. Exp. Med..

[32] Székely J.I., Pataki Á. (2012). Effects of vitamin D on immune disorders with special regard to asthma, COPD and autoimmune diseases: A short review. Experts Rev..

[33] Penna G., Amuchastegui S., Laverny G., Adorini L. (2007). Vitamin D receptor agonists in the treatment of autoimmune diseases: Selective targeting of myeloid but not plasmacytoid dendritic cells. J. Bone Miner. Res..

[34] Motrich R.D., van Etten E., Depovere J., Riera C.M., Rivero V.E., Mathieu C. (2009). Impact of vitamin D receptor activity on experimental autoimmune prostatitis. J. Autoimmun..

[35] Gauzzi M.C., Purificato C., Donato K., Jin Y., Wang L., Daniel K.C., Maghazachi A.A., Belardelli F., Adorini L., Gessani S. (2005). Suppressive effect of 1alpha,25-dihydroxyvitamin D_3_ on type I IFN-mediated monocyte differentiation into dendritic cells: Impairment of functional activities and chemotaxis. J. Immunol..

[36] Lee J.H., Park S., Cheon S., Lee J.H., Kim S., Hur D.Y., Kim T.S., Yoon S.R., Yang Y., Bang S.I. (2011). 1,25-Dihydroxyvitamin D_3_ enhances NK susceptibility of human melanoma cells via Hsp60-mediated FAS expression. Eur. J. Immunol..

[37] El-Shazly A.E., Lefebvre P.P. (2011). Modulation of NK cell autocrine-induced eosinophil chemotaxis by interleukin-15 and vitamin D_3_: A possible NK-eosinophil crosstalk via IL-8 in the pathophysiology of allergic rhinitis. Mediat. Inflamm..

[38] Brandt C.S., Baratin M., Yi E.C., Kennedy J., Gao Z., Fox B., Haldeman B., Ostrander C.D., Kaifu T., Chabannon C. (2009). The B7 family member B7-H6 is a tumor cell ligand for the activating natural killer cell receptor NKp30 in humans. J. Exp. Med..

[39] Simhadri V.R., Reiners K.S., Hansen H.P., Topolar D., Simhadri V.L., Nohroudi K., Kufer T.A., Engert A., Pogge von Strandmann E. (2008). Dendritic cells release HLA-B-associated transcript-3 positive exosomes to regulate natural killer function. PLoS One.

[40] Marras F., Nicco E., Bozzano F., Di Biagio A., Dentone C., Pontali E., Boni S., Setti M., Orofino G., Mantia E. (2013). Natural killer cells in HIV controller patients express an activated effector phenotype and do not up-regulate NKp44 on IL-2 stimulation. Proc. Natl. Acad. Sci. USA.

[41] De Maria A., Biassoni R., Fogli M., Rizzi M., Cantoni C., Costa P., Conte R., Mavilio D., Ensoli B., Cafaro A. (2001). Indentification, molecular cloning and functional characterization of NKp46 and NKp30 natural cytotoxicity receptors in Macaca fascicularis NK cells. Eur. J. Immunol..

[42] Arnon T.I., Achdout H., Lieberman N., Gazit R., Gonen-Gross T., Katz G., Bar-Ilan A., Bloushtain N., Lev M., Joseph A. (2004). The mechanisms controlling the recognition of tumor- and virus-infected cells by NKp46. Blood.

[43] Maghazachi A.A. (2005). Insights into seven and single transmembrane-spanning domain receptors and their signaling pathways in human natural killer cells. Pharmacol. Rev..

[44] Raϊch-Regué D., Grau-López L., Naranjo-Gómez M., Ramo-Tello C., Pujol-Borrell R., Martínez-Cáceres E., Borràs F.E. (2012). Stable antigen-specific T-cell hyporesponsiveness induced by tolerogenic dendritic cells from multiple sclerosis patients. Eur. J. Immunol..

[45] Kappos L., Antel J., Comi G., Montalban X., O’Connor P., Polman C.H., Haas T., Korn A.A., Karlsson G., Radue E.W. (2006). Oral fingolimod (FTY720) for relapsing multiple sclerosis. N. Engl. J. Med..

[46] O’Connor P., Comi G., Montalban X., Antel J., Radue E.W., de Vera A., Pohlmann H., Kappos L. (2009). FTY720 D2201 Study Group: Oral fingolimod (FTY720) in multiple sclerosis: Two-year results of a phase II extension study. Neurology.

[47] Pyne S., Pyne N.J. (2013). New perspectives on the role of sphingosine 1-phosphate in cancer. Handb. Exp. Pharmacol..

[48] Jin Y., Knudsen E., Wang L., Bryceson Y., Damaj B., Gessani S., Maghazachi A.A. (2003). Sphingosine 1-phosphate is a novel inhibitor of T-cell proliferation. Blood.

